# Astrocyte expression of aging-associated markers positively correlates with neurodegeneration in the frontal lobe of the rhesus macaque brain

**DOI:** 10.3389/fnagi.2024.1368517

**Published:** 2024-03-20

**Authors:** Miranda D. Horn, Sophia C. Forest, Ahmad A. Saied, Andrew G. MacLean

**Affiliations:** ^1^Brain Institute, Tulane University, New Orleans, LA, United States; ^2^University of Texas, Austin, TX, United States; ^3^Tulane National Primate Research Center, Covington, LA, United States; ^4^Tulane Center for Aging, New Orleans, LA, United States; ^5^Department of Microbiology and Immunology, Tulane University School of Medicine, New Orleans, LA, United States

**Keywords:** neurodegeneration, non-human primate, healthspan, eugeric aging, cellular senescence, inflammaging

## Abstract

**Introduction:**

As the population over the age of 65 increases, rates of neurodegenerative disorders and dementias will rise – necessitating further research into the cellular and molecular mechanisms that contribute to brain aging. With the critical importance of astrocytes to neuronal health and functioning, we hypothesized that alterations in astrocyte expression of aging-associated markers p16^INK4a^ (p16) and sirtuin 1 (SIRT1) with age would correlate with increased rates of neurodegeneration, as measured by FluoroJade C (FJC) staining.

**Methods:**

To test this hypothesis, 19 rhesus macaques at the Tulane National Primate Research Center were selected based on the following criteria: archival FFPE CNS tissue available to use, no noted neuropathology, and an age range of 5–30 years. Tissues were cut at 5 μm and stained for GFAP, p16, SIRT1, and FJC, followed by whole-slide imaging and HALO® image analysis for percentage of marker-positive cells and relative intensity of each stain.

**Results:**

We found the percentage of p16+ cells increases with age in total cells and astrocytes of the frontal (*p* = 0.0021, *p* = 0.0012 respectively) and temporal (*p* = 0.0226, *p* = 0.0203 respectively) lobes, as well as the relative intensity of p16 staining (frontal lobe: *p* = 0.0060; temporal lobe: *p* = 0.0269). For SIRT1, we found no correlation with age except for an increase in the relative intensity of SIRT1 in the temporal lobe (*p* = 0.0033). There was an increase in neurodegeneration, as measured by the percentage of FJC+ cells in the frontal lobe with age (*p* = 0.0057), as well as in the relative intensity of FJC staining in the frontal (*p* = 0.0030) and parietal (*p* = 0.0481) lobes. Importantly, increased p16 and SIRT1 expression in astrocytes correlated with increasing neurodegeneration in the frontal lobe (*p* = 0.0009, *p* = 0.0095 respectively).

**Discussion:**

Together, these data suggest that age-associated alterations in astrocytes contribute to neurodegeneration and provide a target for mechanistic studies in the future.

## 1 Introduction

As the population ages, the rates of neurodegenerative disorders and dementia increase, with the number of individuals worldwide living with dementia expected to triple by 2050 (World Health Organization, 2022). With this comes an increasing need to understand the mechanisms that differentiate healthy and pathological brain aging. Many hallmarks of brain aging have been well established, including decreases in neurocognitive function that have been associated with decreases in the volume of specific gray and white matter regions and white matter function (Harada et al., 2013). However, the underlying cellular and molecular mechanisms that contribute to these changes have yet to be elucidated. Over the last couple decades many cellular and molecular signatures of aging have been identified, including accumulated DNA damage, altered metabolic signaling, mitochondrial dysfunction, decreased autophagy, and cellular senescence (López-Otín et al., 2013). However, the cell-type and regional variability in these markers of aging across the lifespan is still unclear, especially in the non-human primate (NHP) brain. The completion of this work in NHPs will provide a foundation for future studies into disease processes that may alter the aging timeline in the NHP brain, such as HIV, COVID, and Alzheimer’s Disease to name a few. Additionally, due to their genetic and physiological similarity to humans, NHPs serve as an invaluable resource in studying the aging process in the human brain (Didier et al., 2016). Establishing an understanding of how various markers of aging change in specific cell types and brain regions will aid in the identification of alterations in the aging process that contribute to pathology and facilitate the development of more targeted therapeutics.

Due to the critical role astrocytes play in maintaining the proper functioning of neurons and homeostasis of the brain microenvironment, any alterations in their function with age likely contribute to the dysfunction and degradation of other cell types over time, as has been shown in astrocyte-neuron co-cultures (Pertusa et al., 2007; Kawano et al., 2012). An increase in the astrocyte marker glial fibrillary acid protein (GFAP) – commonly associated with astrogliosis – occurs with increasing age, however, results vary based on the brain regions and species assessed (Nichols et al., 1993; David et al., 1997; Diniz et al., 2010; Kanaan et al., 2010; Rodríguez et al., 2014). Additionally, quantitative measures of alterations in astrocyte morphology have suggested an increase in astrocyte complexity around middle-age that decreases with advanced age to levels that do not differ from that of young animals (Kanaan et al., 2010; Cerbai et al., 2012; Diniz et al., 2016; Robillard et al., 2016). Based on recent advances in the understanding of astrocyte diversity throughout the brain and across the lifespan (Khakh and Sofroniew, 2015; Batiuk et al., 2020), the use of additional markers is necessary to provide a more complete picture of the molecular changes that may be impacting astrocyte functioning with age. We hypothesize that astrocytes in different brain regions will show molecular signs of aging at different rates – with areas where astrocytes are most impacted having the greatest rates of neurodegeneration based on the importance of astrocytes to neuronal health.

To test this hypothesis, we assessed two aging-associated markers and the neurodegeneration marker FluoroJade C (FJC). The cell-cycle arrest protein, p16^INK4a^ (henceforth referred to as p16), is one of the most well-studied markers of cellular senescence (Krishnamurthy et al., 2004; Hernandez-Segura et al., 2018; Gorgoulis et al., 2019). Within human frontal cortex, p16 expression has been shown to increase in astrocytes with normal aging (Bhat et al., 2011); however, regional brain expression of p16 with age has not been assessed. The second marker we chose to assess was the NAD-dependent deacetylase sirtuin 1 (SIRT1), which decreases as a result of DNA damage and is critical to metabolic functioning (Kubben and Misteli, 2017). SIRT1 is well supported as an ‘anti-aging’ protein, with many studies showing that increasing its activity can block or even reverse signs of aging (Kubben and Misteli, 2017; Lee et al., 2019). However, little is known about the expression of SIRT1 in astrocytes across the lifespan. Here, we utilize archival brain tissues from rhesus macaques to assess the distribution of p16 and SIRT1 expression in astrocytes across the lifespan and the association of these aging markers with neurodegeneration.

## 2 Materials and methods

### 2.1 Ethics statement, animal housing, and selection of tissues

Animals on this study were colony reared and housed outdoors in family troops within large corals. Practices in the housing and care of animals conformed to the regulations and standards of the U.S. Department of Health and Human Services Public Health Service (PHS) Policy on Humane Care and Use of Laboratory Animals, and the Guide for the Care and Use of Laboratory Animals. The Tulane National Primate Research Center (TNPRC; Animal Welfare Assurance # A4499-01) is fully accredited by the Association for the Assessment and Accreditation of Laboratory Animal Care-International. All animals are routinely cared for according to the guidelines prescribed by the NIH Guide to Laboratory Animal Care. The TNPRC conducts all research in accordance with the recommendations of the Weatherall report - “The use of non-human primates in research.” The Institutional Animal Care and Use Committee (IACUC) of the TNPRC approved all animal-related protocols, including any treatments used with non-human primates. All animal procedures were overseen by veterinarians and their staff.

Any animals housed indoors were maintained in Animal Biosafety Level 2 housing with a 12:12-h light: dark cycle, relative humidity 30–70%, and a temperature of 17.8–28.9°C. Water was available *ad libitum*, and a standard commercially formulated non-human primate diet (Lab Fiber Plus Monkey DT, 5 K63, PMI Nutrition International, St. Louis, MO) was provided twice daily and supplemented daily with fresh fruit and/or forage material as part of the environmental enrichment program. All animals at the TNPRC have environmental enrichment, widely used to improve welfare in captive macaques.

Animals were humanely euthanized by the veterinary staff at the TNPRC in accordance with endpoint policies. Euthanasia was conducted by anesthesia with ketamine hydrochloride (10 mg/kg) followed by an overdose with sodium pentobarbital and immediate necropsy. This method was consistent with the recommendation of the American Veterinary Medical Association guidelines. Four brain regions approximately 1 cm thick are routinely collected during necropsy of colony animals at the TNPRC representing frontal lobe, parietal lobe, temporal lobe, and cerebellum. All tissues are fixed at routine necropsy by immersion in 10% neutral buffered formalin with zinc modification for 48 h before trimming and paraffin embedding.

To investigate the relative expression of aging markers, 19 rhesus macaques (*Macaca mulatta*) were used. Three age ranges were established for the selection of animals: 5–10 years, 11–20 years, and greater than 20 years old to ensure distribution of animals across the adult lifespan. Male and female macaques were randomly selected based on the following criteria: age range from 5 to 30 years old and lack of noted neuropathology. The selected animals were colony animals that were presented for necropsy due to a variety of natural disease processes that include gastroenteritis, weight loss, arthritis, and amyloidosis. Additionally, the animals had brain tissues collected at necropsy, preserved in formalin and available for the aging markers evaluation.

### 2.2 Immunofluorescent staining

Tissue samples were initially formalin-fixed and paraffin-embedded (FFPE). Subsequently, 5-micrometer-thick sections were prepared using an ultramicrotome. Prior to staining, slides were incubated overnight at 60°C to enhance tissue adhesion. The sections then underwent a deparaffinization process with xylene, followed by rehydration through an ethanol and deionized water gradient. Antigen unmasking was achieved using a 1% low pH antigen retrieval solution (Antigen Unmasking Solution, Citric Acid Based, Vector Laboratories, Inc., cat. #H-3300) heated to boiling, with slides incubated in the solution for an hour as the solution cooled to room temperature. Slides were rinsed in a 1X Phosphate Buffered Solution with 0.2% Fish Skin Gelatin (PBS-FSG) wash buffer prior to application of blocking agent for 40 min (normal goat serum, MP Biomedicals, LLC., cat. #2939149; or normal donkey serum, GeminiBio, cat. #100–151). For p16 staining, anti-p16^INK4a^ (Invitrogen, Ms. Mab IgG1, MA5-17093, 1:100) was applied for 1 h at room temperature. For SIRT1 staining, anti-SIRT1 (Lifespan Biosciences, Inc., Gt Pab, cat. #LS-B8356, 1:100) was applied overnight at 4°C. Secondary antibodies (Goat anti-Mouse IgG (H + L), Alexa Fluor™ 633, Invitrogen, cat. #A-21052; Donkey anti-Goat IgG (H + L) Cross-Adsorbed Secondary Antibody, Alexa Fluor™ 568, Invitrogen, cat. #A-11057) and a conjugated anti-GFAP (Anti-Glial Fibrillary Acidic Protein (GFAP) − Cy3™ antibody, Sigma-Aldrich®, Ms. Mab, cat. #C9205, 1:300; GFAP Monoclonal Antibody (GA5), Alexa Fluor™ 488, eBioscience™, Ms. Mab, cat. #53–9,892-82, 1:300) were applied for 1 h at room temperature. Slides were washed twice in 1X PBS-FSG with 0.1% Triton-100 for 5 min and once in 1X PBS-FSG for 5 min after each antibody application. Finally, slides were washed for 5 min in 1X PBS prior to adding 20 μL of EverBrite TrueBlack® Hardset Mounting Medium (Biotium, cat. #23018) and a coverslip. Slides were left to dry overnight at room temperature before being imaged.

### 2.3 FluoroJade C staining

Slides containing 5 μm thick sections of FFPE tissue were incubated overnight at 60°C to melt wax and ensure optimum tissue adhesion. Slides were then deparaffinized by incubating three times in xylene for 5 min each, followed by one 3-min incubation in 100% ethanol, one 3.5-min incubation in 100% ethanol, one 3-min incubation in 95% ethanol, and one 5-min incubation in 80% ethanol. Slides were then treated according to instructions for the Fluoro-Jade C Staining Kit with DAPI Counter Stain (Histo-Chem Inc., cat. #FJC-SK-DAPI). Briefly, slides were incubated for 2 min in 70% ethanol followed by deionized water prior to incubation in potassium permanganate for 5 min. Slides were rinsed twice in deionized water for 2 min prior to incubation in FluoroJade C and DAPI mixture for 10 min. Slides were then rinsed three times in deionized water before being dried in a slide warmer at 60°C for 15 min. Finally, slides were cleared by a 1-min incubation in xylene and coverslipped using Micromount mounting media (Leica Biosystems, cat. #3801730) and left overnight to dry before imaging.

### 2.4 Slide imaging, analysis, and statistics

All slides were scanned using a Zeiss Axio Scan.Z1 slide scanner at 20x magnification. The entire tissue was selected for scanning and scan settings were optimized for each brain region and stain. An empty channel was included in the scan for analysis of autofluorescent signal. Scans were imported into the HALO® Image Analysis software (Indica Labs) and were analyzed using the fluorescent object colocalization module of the HALO® Image Analysis software (Indica Labs). Summary data was exported as an Excel sheet that included the percentage of marker (p16, SIRT1, or FJC) positive cells. For the percentage of marker positive astrocytes, the number of co-labeled cells was divided by the total number of GFAP+ cells and multiplied by 100. For all analyses, autofluorescent cells (cells positive for all fluorescent channels) were removed. For marker intensity, the average cell intensity for the marker was divided by the average cell intensity for DAPI. Correlations were determined by calculating Pearson’s correlation coefficients using Prism GraphPad 10. *p*-values were two-tailed and a value of *p* < 0.05 was considered significant for all analyses.

## 3 Results

### 3.1 GFAP expression increases with age

**Figure 1:**
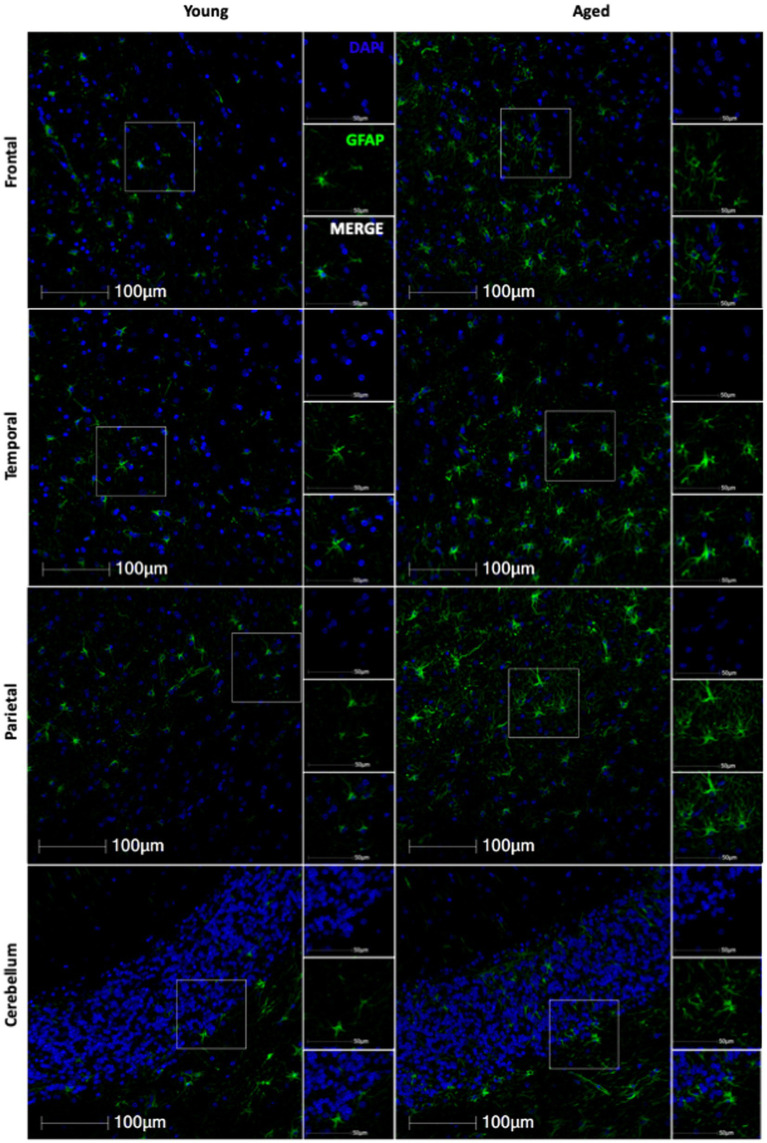
Representative immunofluorescent images of GFAP staining from young and aged animals for each brain region.

**Figure 2:**
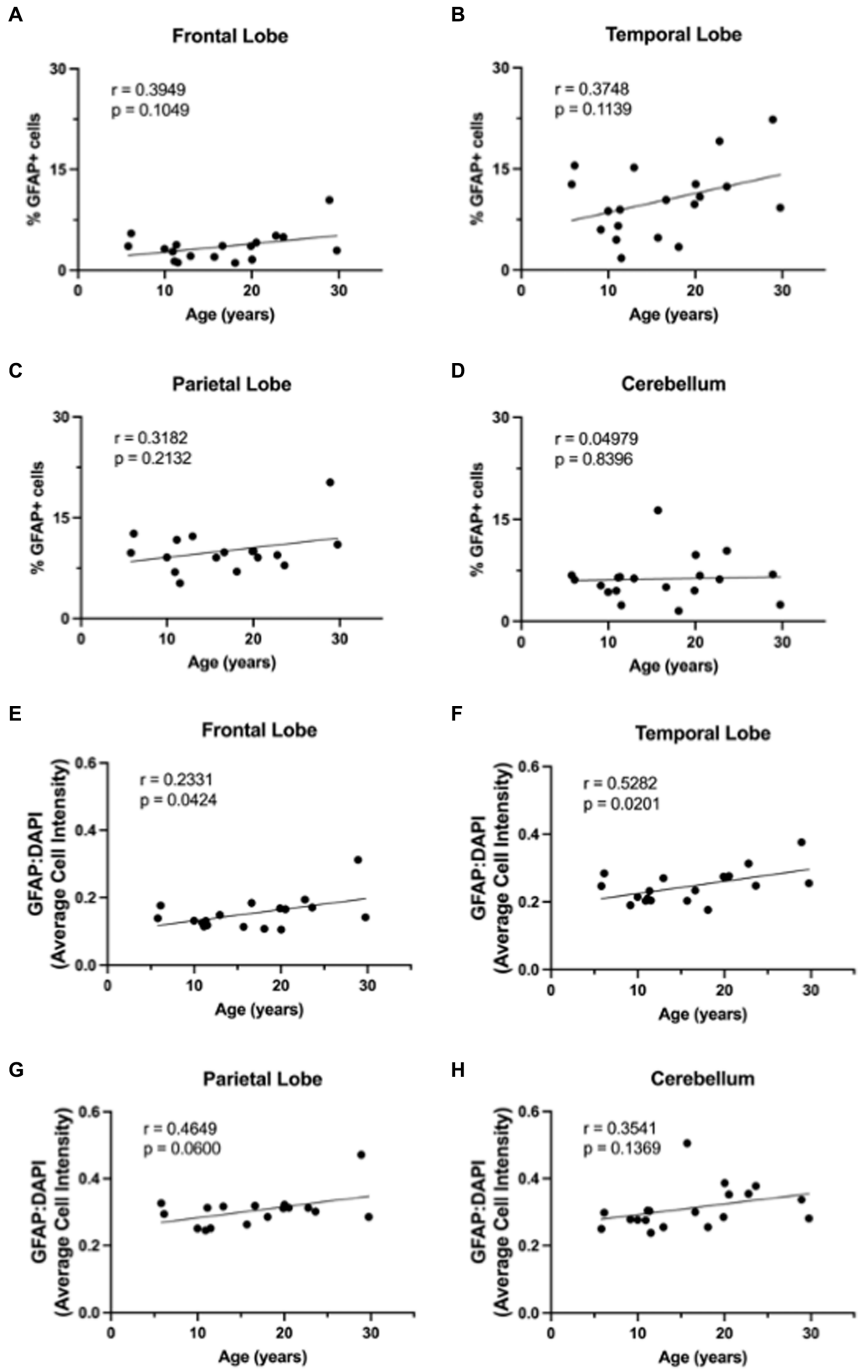
GFAP expression across the lifespan. Percentage of GFAP+ cells across the lifespan generally increases in the frontal lobe **(A)**, temporal lobe **(B)**, parietal lobe **(C)**, but not in the cerebellum **(D)**. Relative intensity of GFAP staining generally increased with age across all brain regions and this correlation was statistically significant in the frontal **(E)** and temporal **(F)** lobes, but not in the parietal lobe **(G)** or cerebellum **(F)**. Pearson correlation coefficients with two-tailed *p* analyses.

Based on the importance of astrocytes to neuronal function, we hypothesized that molecular aging of astrocytes would increase neurodegeneration. To evaluate the effects of healthy aging in astrocytes on neurodegeneration, we selected 19 rhesus macaques from the TNPRC animal colony that had no observable neuropathology and for whom FFPE brain sections were available for use (For animal information, see Table 1). We first assessed GFAP expression to assess the variation of expression across the different brain regions with age. Slides from four routinely collected brain regions were cut and immunofluorescently stained. We then obtained whole-slide images and used HALO® image analysis to assess the percentage of GFAP+ cells and the relative intensity of GFAP staining. While we found an overall increase in GFAP staining with age (Figure 1), the percentage of GFAP+ cells was not significantly correlated with age in any brain region analyzed (Figures 2A–D). However, the relative intensity of GFAP staining was significantly correlated with age in the frontal (Figure 2E; *p* = 0.0424) and temporal (Figure 2F; *p* = 0.0201) lobes and trended toward statistical significance in the parietal lobe (Figure 2G; *p* = 0.0600) and cerebellum (Figure 2H; *p* = 0.1369). Based on these results, we believe the relative intensity of GFAP to be more reliable than the percentage of GFAP+ cells in measuring changes in GFAP expression with age. Additionally, while the relative number of GFAP+ cells does not change with age, the amount of GFAP within cells increases, suggesting that astrocyte aging is associated with structural alterations that likely impact function.

**Table 1:** Animal information.

**Animal ID**	**Age (years)**	**Sex**	**Cause of death**	**Brain regions analyzed**
MG08	5.81	F	Colitis	F, T, P, C
LP22	6.13	F	Generalized amyloidosis	F, T, P, C
KM52	9.14	M	Gastritis	T, C
KH73	9.98	F	Renal failure/Uremia	F, T, P, C
JT82	10.91	F	Generalized amyloidosis	F, T, P, C
JB99	11.13	M	Arthritis	F, T, P, C
JP27	11.34	M	Investigator initiated – Vaccine study	F, T, C
JG69	11.50	M	Intestinal amyloidosis	F, T, P, C
HN93	12.96	F	Colitis	F, T, P, C
HG52	15.70	M	Colitis	F, T, P, C
FH89	16.63	F	Endometriosis	F, T, P, C
FE85	18.10	F	Colitis	F, T, P, C
EA40	19.90	F	Generalized amyloidosis	F, T, P, C
DG36	20.03	F	Dermatitis	F, T, P, C
CM82	20.52	F	Trauma – Monkey bite	F, T, P, C
NK84	22.77	F	Euthanasia – Progressive hyperglycemia	F, T, P, C
BK47	23.62	F	Generalized amyloidosis	F, T, P, C
M791	28.90	M	Arthritis	F, T, P, C
N026	29.76	F	Generalized amyloidosis	F, T, P, C

### 3.2 Cellular senescence marker p16 increases with age

**Figure 3:**
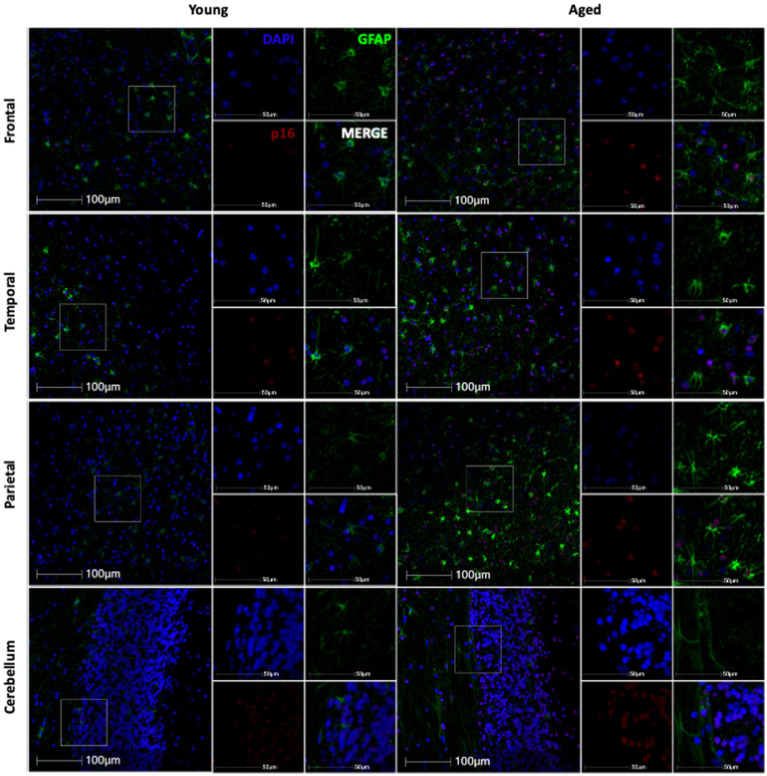
Representative immunofluorescent images of p16 staining from young and aged animals for each brain region.

**Figure 4:**
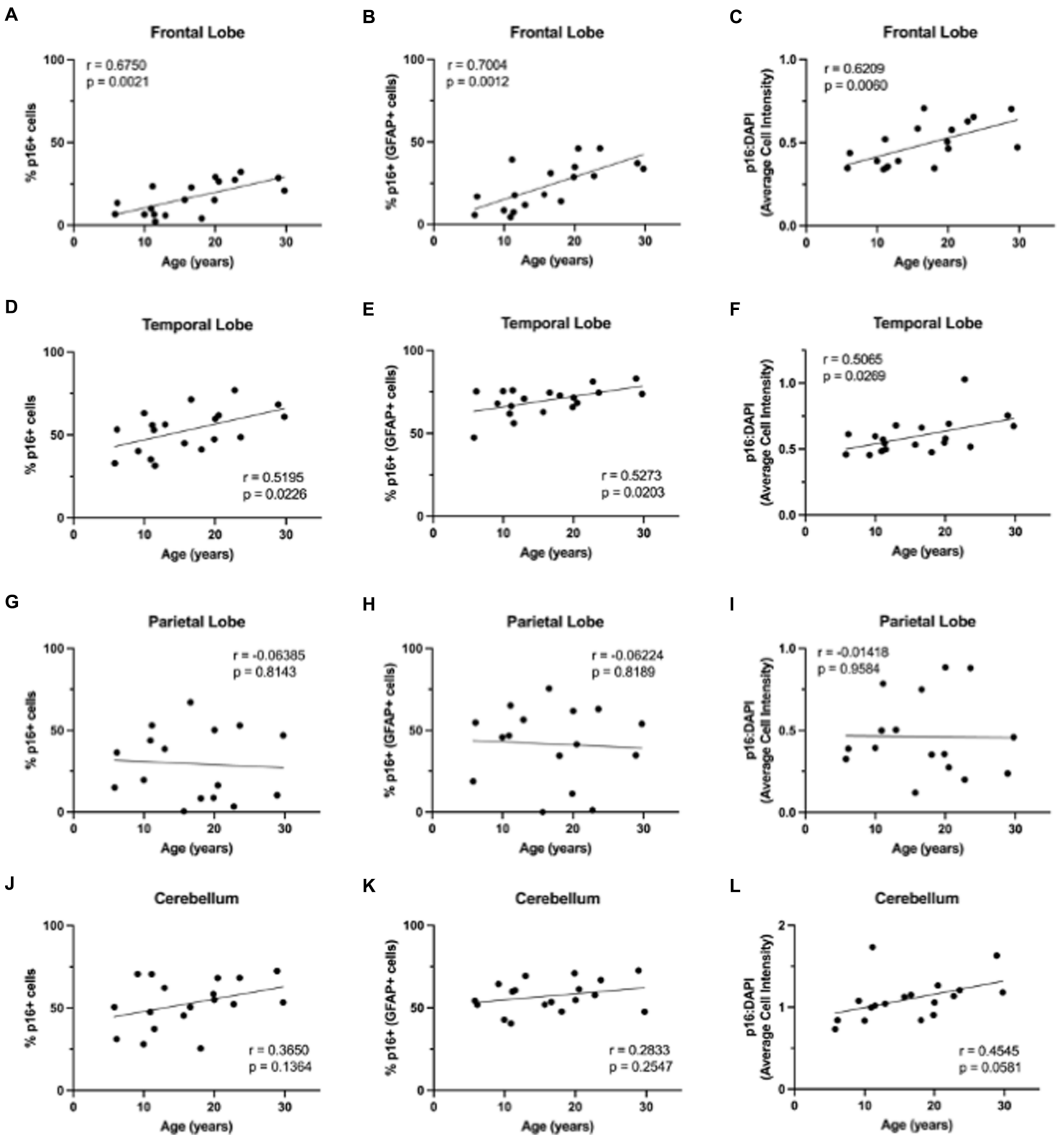
Expression of p16INK4a increases with age in the brains of rhesus macaques. p16INK4a is positively correlated with age in total cells, astrocytes, and overall relative intensity in the frontal **(A–C)** and temporal **(D–F)** lobes. No correlations were found for p16INK4a expression in the parietal lobe **(G–I)** or cerebellum **(J–L)**. Pearson correlation coefficients with two-tailed *p* analyses.

Next, we wanted to determine if non-human primate astrocytes demonstrated molecular markers of aging. We began by looking at p16, which we hypothesized would increase in astrocytes with increasing age. To determine if p16 increased with age, we stained slides from each brain region for DAPI, p16, and GFAP. Using whole-slide imaging and HALO® image analysis, we assessed the percentage of p16 positive cells, p16 positive astrocytes, and the relative intensity of p16 staining in each brain region. In general, we saw an increase in p16 expression with age (Figure 3). Specifically, we found an increase in the percentage of p16-positive total cells and astrocytes of the frontal (Figure 4A; *p* = 0.0021; Figure 4B; *p* = 0.0012) and temporal (Figures 4D; *p* = 0.0226; Figure 4E; *p* = 0.0203) lobes. We also saw an overall increase in the relative intensity of p16 staining in each of these brain regions (Figure 4C; *p* = 0.0060; Figure 4F; *p* = 0.0269). This same pattern was observed in the cerebellum but did not reach statistical significance (Figure 4J–L). In contrast, no correlation was found between p16 expression and age in the parietal lobe (Figures 4G–I), where there was considerably more variability in p16 expression between animals. These data demonstrate that p16 is a reliable marker of aging in total cells and astrocytes of the frontal and temporal lobes. However, the lack of significant correlation between p16 expression and aging in the cerebellum and parietal lobes suggests that astrocytes in these regions may be more resistant to aging or may age via alternative molecular mechanisms.

### 3.3 SIRT1 expression in astrocytes does not change with age

**Figure 5:**
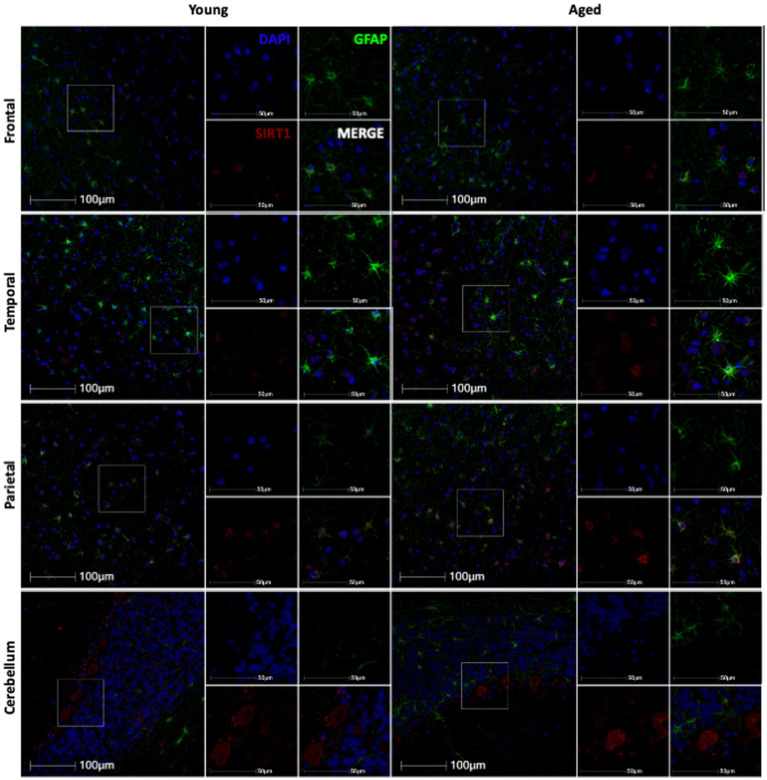
Representative immunofluorescent images of SIRT1 staining from young and aged animals for each brain region.

**Figure 6:**
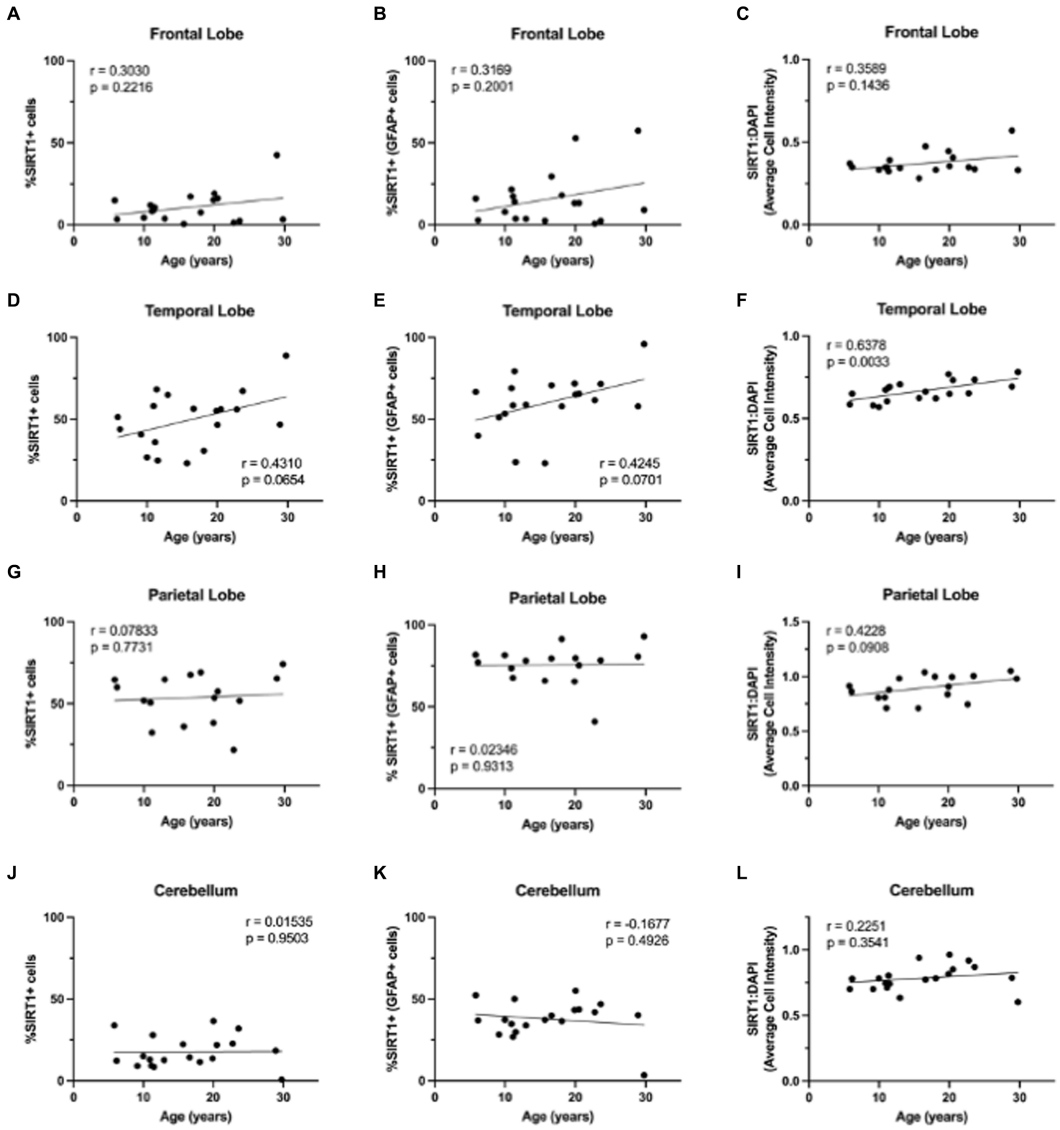
Expression of SIRT1 does not change with age in the brains of rhesus macaques. The only significant correlation between SIRT1 and age was in the relative intensity of SIRT1 in the temporal lobe **(F)**. No significant correlations were found for SIRT1 expression in the frontal lobe **(A–C)**, total cells or astrocytes of the temporal lobe **(D, E)**, parietal lobe **(G–I)**, or cerebellum **(J–L)**. Pearson correlation coefficients with two-tailed *p* analyses.

While p16 expression is a wide-used measure of cellular senescence, it represents only one potential mechanism of cellular aging. To assess the aging mechanisms of astrocytes more broadly, we also assessed the expression of SIRT1. As SIRT1 has been shown to decrease with age in numerous cell types, especially in association with cellular senescence (Xu et al., 2020), we expected to see a decrease in SIRT1 with age in both the total cell population and astrocytes. To determine the expression of SIRT1 in the brain throughout the lifespan of non-human primates, we immunofluorescently stained slides for DAPI, SIRT1, and GFAP. Here, whole-slide imaging and HALO® image analysis revealed a general increase of SIRT1 expression in total cells and astrocytes of the frontal and temporal lobes with age (Figure 5), but these trends were not statistically significant (Figures 6A–E) other than for the relative intensity of SIRT1 in the temporal lobe (Figure 6F; *p* = 0.0033). In the cerebellum and parietal lobes there was no apparent relationship between SIRT1 expression and age (Figure 5 and Figures 6G–L). We conclude that SIRT1 expression does not decrease with age in the total cell population nor in astrocytes across any of the brain regions tested. Based on these results, SIRT1 expression alone does not appear to be a reliable marker of aging in astrocytes.

### 3.4 Neurodegeneration increases with age

**Figure 7:**
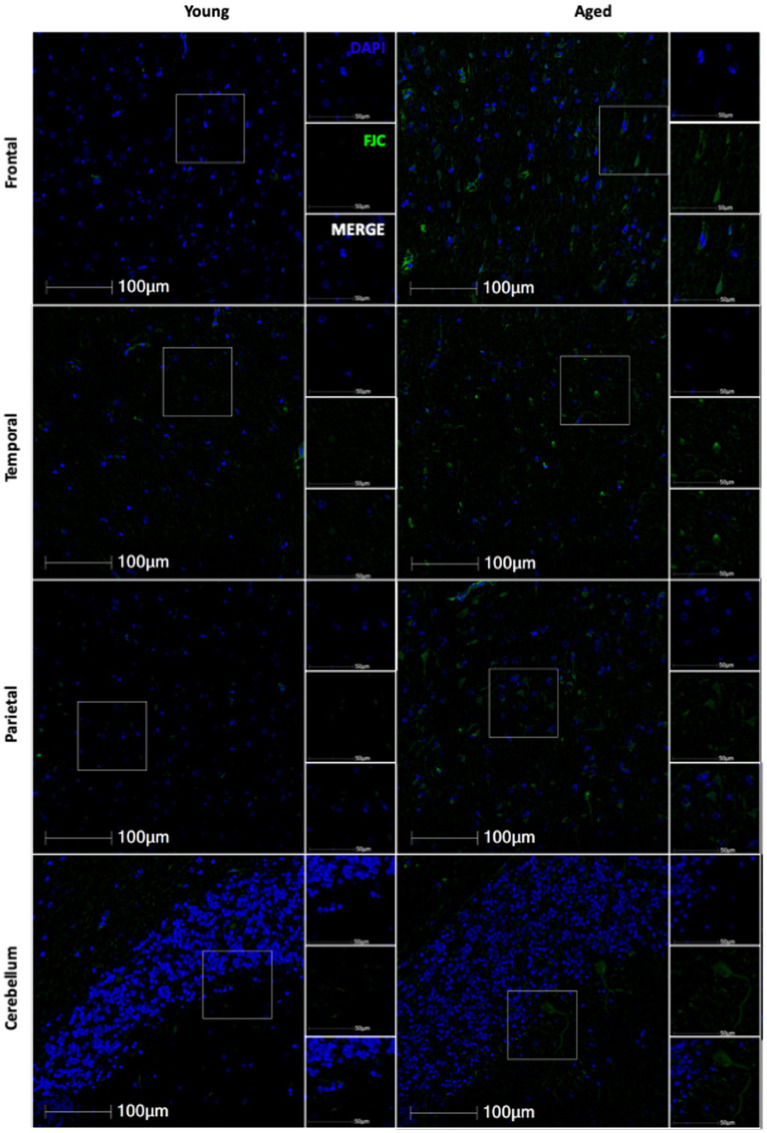
Representative images of FJC staining across several brain regions in young and aged animals.

**Figure 8:**
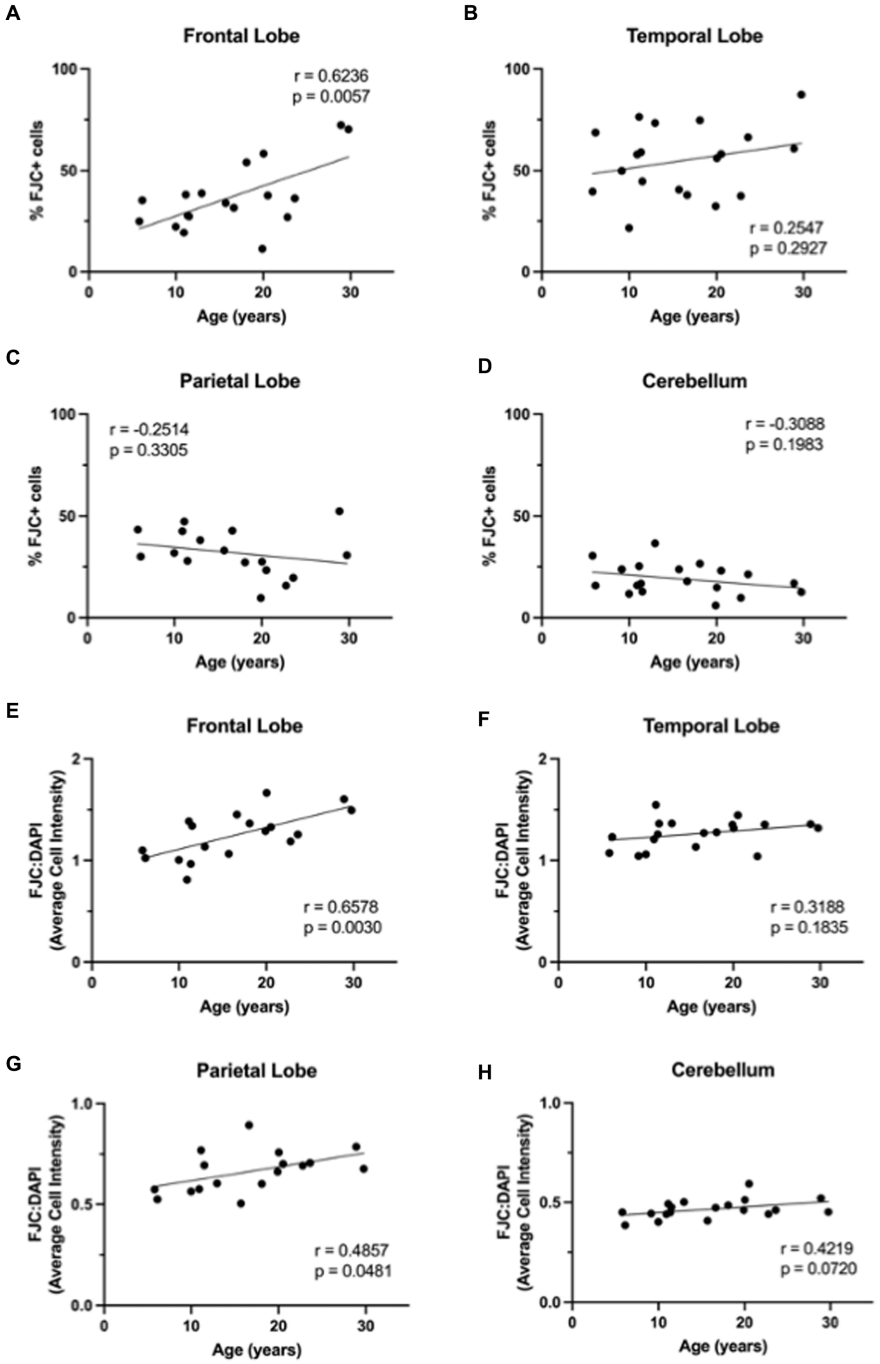
FluoroJade C (FJC) staining increases with age in rhesus macaques. The %FJC+ cells increased significantly with increasing age in the frontal lobe **(A)**, but no correlations were found between %FJC+ cells and age in the temporal lobe **(B)**, parietal lobe **(C)**, or cerebellum **(D)**. The intensity of FJC staining relative to DAPI staining generally increased with age across all brain regions **(E–H)** and was significantly correlated with age in the frontal **(E)** and parietal **(G)** lobes. Pearson correlation coefficients with two-tailed *p* analyses.

We next wanted to determine if neurodegeneration increased with age in this cohort of animals. To assess generalized neurodegeneration throughout the brain and across the lifespan, we performed FJC staining for each brain region. In this cohort of animals, we found the correlation between FJC staining and age varied by brain region (Figure 7), with an increase in the percentage of FJC+ cells in the frontal and temporal lobes (Figures 8A,B) and a decrease in the percentage of FJC+ cells in the parietal lobe and cerebellum (Figures 8C,D). However, the only statistically significant correlation between percentage of FJC+ cells and age was in the frontal lobe where FJC staining increased with age (Figure 8A; *p* = 0.0057). When looking at the intensity of FJC staining relative to DAPI staining, there was a general increase with age across all brain regions (Figures 8E–H), with a statistically significant positive correlation with age in the frontal (Figure 8E; *p* = 0.0030) and parietal (Figure 8G; *p* = 0.0481) lobes and a trend toward significance in the cerebellum (Figure 8H; *p* = 0.0720). These results suggest the frontal and parietal lobes are the most sensitive to the damaging effects of aging in non-human primates. Importantly, intensity of FJC staining appears to be a more reliable indicator of neurodegeneration with age than the percentage of FJC+ cells.

### 3.5 Neurodegeneration correlates with aging markers in astrocytes of the frontal lobe

**Figure 9:**
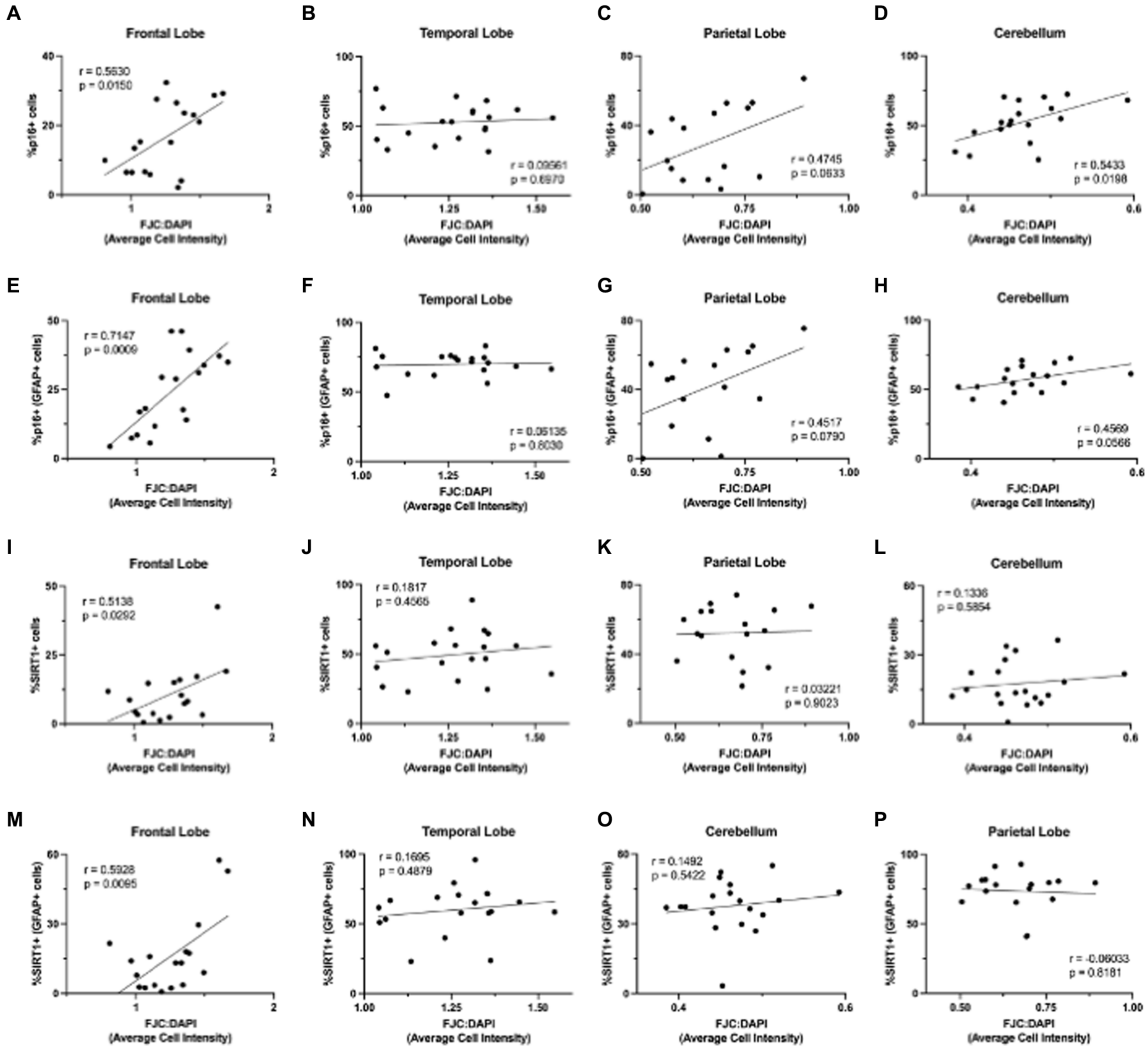
Neurodegeneration increased with increasing expression of aging markers. Neurodegeneration, as measured by FJC staining, increased with increasing p16INK4a (p16) expression **(A–H)**, with significant positive correlations in total cells of the frontal lobe **(A)** and cerebellum **(C)**, and astrocytes of the frontal lobe **(E)**. The relationship between neurodegeneration and SIRT1 was less pronounced **(I–P)**, but still showed significant positive correlations in total cells **(I)** and astrocytes **(M)** of the frontal lobe. Pearson correlation coefficients with two-tailed *p* analyses.

Finally, we were interested in whether p16 and SIRT1 expression correlated with neurodegeneration, as measured by FJC staining. To determine the relationship between p16 and neurodegeneration, we calculated the Pearson correlation coefficient between the percentage of p16+ cells (in astrocytes and total cells) and FJC intensity (Figures 9A–H). Neurodegeneration generally increased with increasing p16 in all brain regions except the temporal lobe. This correlation was statistically significant in the total cells of the frontal lobe (Figure 9A; *p* = 0.0150) and cerebellum (Figure 9D; *p* = 0.0198) as well as in astrocytes of the frontal lobe (Figure 9E; *p* = 0.0009). We repeated this for the percentage of SIRT1+ cells (in astrocytes and total cells) and FJC intensity (Figures 9I–P). Here, there was a positive correlation between SIRT1 and neurodegeneration only in the frontal lobe (Figure 9I,M). This correlation was statistically significant for both the total cell population (Figures 9I; *p* = 0.0292) and astrocytes (Figure 9M; *p* = 0.0095). It is important to note that the significance of the SIRT1 results were largely driven by a single aged animal (M791), that was euthanized due to severe arthritis – a disease of eugeric aging. Multiple linear regression analyses were also performed to assess the interaction between age, p16, and SIRT1 in predicting neurodegeneration. Here, only age and the interaction between age and SIRT1 were significant (*p* = 0.04145 and *p* = 0.0333, respectively). Together, these results demonstrate the relevance of p16 and SIRT1 expression in astrocytes to neurodegeneration of the frontal lobe.

## 4 Discussion

As the human population ages, it is critical to increase our understanding of molecular mechanisms differentiating pathological from non-pathological aging. This knowledge will guide more targeted prevention and therapeutic strategies to increase the human healthspan. Within the brain, we must assess mechanisms underlying cellular aging across cell types and how those changes impact neurodegeneration to better understand the pathophysiology of cognitive decline and dementia. As astrocytes are critical to the health and function of neurons, they are an obvious starting point when looking for cells that may impact neurodegeneration.

In this study, we found that while the number of GFAP+ astrocytes does not change with age, the overall expression of GFAP increases, suggesting morphological alterations occur (Figures 1, 2). While morphological analysis was not done in this study, in a separate cohort of animals, we have previously shown that the complexity of astrocytes increases between young and adult animals, but that increase is lost in geriatric animals (Robillard et al., 2016). Additionally, the lack of a change in number of GFAP+ cells ensures that we are not seeing more p16+ or SIRT1+ astrocytes simply because we are detecting more astrocytes with age. Though it is important to note the limitations of GFAP as a pan-astrocyte marker, as astrocytes are highly heterogeneous throughout the brain and certain subtypes express GFAP at very low levels or not at all (Molofsky et al., 2012; Batiuk et al., 2020).

When looking at p16, a cell-cycle arrest protein associated with cellular senescence, we found that it increased with age in the frontal and temporal lobes in both the total cell population and in astrocytes (Figures 3, 4A–F). Importantly, the expression of p16 was correlated with increasing neurodegeneration in the frontal lobe and cerebellum (Figures 9A,D,E), suggesting the importance of this marker in the damaging effects of aging in these regions. As chronically senescent cells are generally dysfunctional and known to have damaging effects on nearby cells via their senescence-associated secretory phenotype (van Deursen, 2014; Tripathi et al., 2021; Huang et al., 2022), these data also suggest an important role of astrocyte senescence in the frontal lobe and support the potential for senolytics as a therapeutic strategy in combating neurodegeneration (Bussian et al., 2018).

In our assessment of SIRT1 expression, we found an increase with age in the temporal lobe only (Figures 5, 6F) and only found a correlation between SIRT1 and neurodegeneration in the frontal lobe (Figures 9I,M). While we expected to see a decrease in SIRT1 expression, as SIRT1 overexpression has been associated with extended lifespan and decreased cellular senescence (Lee et al., 2019), we saw no change with age, or even an increase in expression. This may be explained by the fact that there has been some evidence that the relationship between SIRT1 expression and aging varies by tissue type (Xu et al., 2020) and the beneficial effects of overexpression may be dose-dependent, with high levels being detrimental (Alcendor et al., 2007; Whitaker et al., 2013). Importantly, a study looking at the expression of sirtuins in the rat brain found increased levels of SIRT1 mRNA and protein across several brain regions, but found decreased activity of SIRT1 in these same regions and an associated increase in the acetylation of p53 (Braidy et al., 2015). This suggests that SIRT1 activity may be more important than expression alone in mediating its anti-aging effects.

Finally, we found a general increase in neurodegeneration with age across all brain regions (Figures 7, 8E–H). Together, this data highlights the variable impacts of aging across the brain and how certain markers of aging may be of more importance than others depending on the brain region of interest. Further, the frontal lobe was the most sensitive to aging with the greatest correlation between aging markers and neurodegeneration of any brain region analyzed. This parallels human data and supports the frontal aging hypothesis (West, 1996, 2000), which posits that the cognitive functions most vulnerable to aging are mediated by the frontal lobe.

While this data provides a strong foundation for understanding the effects of aging in astrocytes throughout the NHP brain, there are a few limitations to consider. For one, while these animals were free of any signs of neurological disease at the time of death, they were euthanized due to clinical conditions that may have impacts on neuroinflammation and neurodegeneration. For example, several animals were euthanized due to gastrointestinal issues and likely had severe dysbiosis of their gut microbiota, which has been linked to several neurodegenerative disorders (Peterson, 2020). Additionally, while tissue sections were from the same broad region of the brain, there was considerable variability in the precise subregions between animals, especially for regions other than the frontal lobe. This may account for the limited correlation between aging markers and age in the temporal lobe, parietal lobe, and cerebellum and additional studies with more conserved tissue sections are needed. There are also known sex differences in the effects of aging on the human brain (Salwierz et al., 2022) that this study was not powered to assess. Importantly, the majority of animals in this study were female which may explain the limited expression of aging markers and neurodegeneration as females have longer lifespans (Lemaître et al., 2020) and slower metabolic aging (Goyal et al., 2019) than males. This is especially interesting when considering the SIRT1 results, where the significant correlation with neurodegeneration in the frontal lobe was largely driven by the one male over the age of 20. Finally, the total cell population showed increased expression of p16 and SIRT1 in the frontal lobe, where GFAP+ astrocytes only accounted for approximately 5–10% of the cellular populations, suggesting that these markers are likely elevated in other cell types as well. Further studies assessing these markers in microglia, neurons, oligodendrocytes, and endothelial cells are needed to gain a deeper understanding of the cell types most impacted and begin to discern the cellular mechanisms underlying neurodegeneration and cognitive decline.

Our results highlight the importance of studying molecular aging markers across multiple cell types and brain regions to develop a deeper understanding of the cell- and region-specific molecular changes contributing to neurodegeneration. Also, it demonstrates the sensitivity of the frontal lobe to the detrimental effects of molecular aging and supports further focus on this brain region in analysis of cellular and molecular mechanisms associated with cognitive decline. Future studies that combine assessment of aging markers in the frontal lobe with cognitive abilities will further discern the causative nature of these markers in the progression of neurodegeneration and dementia.

## Data availability statement

The raw data supporting the conclusions of this article will be made available by the authors, without undue reservation.

## Ethics statement

The animal study was approved by Tulane National Primate Research Center. The study was conducted in accordance with the local legislation and institutional requirements.

## Author contributions

MH: Conceptualization, Formal analysis, Investigation, Methodology, Supervision, Visualization, Writing – original draft, Writing – review & editing. SF: Formal analysis, Investigation, Writing – original draft. AS: Formal analysis, Investigation, Methodology, Writing – review & editing. AM: Conceptualization, Formal analysis, Funding acquisition, Methodology, Project administration, Supervision, Writing – original draft, Writing – review & editing.
